# Long noncoding RNA LINC01106 promotes lung adenocarcinoma progression via upregulation of autophagy

**DOI:** 10.32604/or.2024.047626

**Published:** 2024-12-20

**Authors:** GENGYUN SUN, YIPING ZHENG, JIANFENG CAI, JIE GAO, LIE DONG, XIANGBIN ZHANG, YINGHUI HUANG

**Affiliations:** 1 Department of Respiratory and Critical Care Medicine, The First Affiliated Hospital of Anhui Medical University, Hefei, 230022, China; 2 Department of Respiratory and Critical Care Medicine, Nanping First Hospital Affiliated to Fujian Medical University, Nanping, 353006, China

**Keywords:** LINC01106, TAF15, TEAD4, ATG5, Lung adenocarcinoma (LUAD), Non-small cell lung cancer (NSCLC)

## Abstract

**Background:**

Long noncoding RNA, LINC01106 exhibits high expression in lung adenocarcinoma (LUAD) tumor tissues, but its functional role and regulatory mechanism in LUAD cells remain unclear.

**Methods:**

LINC01106 expression was analyzed in LUAD tissues and its functional impact on LUAD cells was assessed. LUAD cells were silenced with sh-LINC01106 and injected into nude mice to investigate tumor growth. The downstream transcription factors and molecular mechanism were determined using the Human transcription factor database (TFDB) database and Gene Expression Profiling Interactive Analysis (GEPIA) database. Additionally, the impact of linc01106 on autophagy was analyzed by determining the expression of autophagy-related genes (ATGs) in LUAD cells.

**Results:**

Our results showed that LINC01106 exhibited upregulation in both LUAD tissues and cell lines. The silencing of LINC01106 demonstrated a suppressive effect on tumorigenesis in a xenograft mouse model of LUAD. Additionally, LINC01106 was found to recruit TATA-binding protein-associated factor 15 (TAF15), an RNA-binding protein, thereby enhancing the mRNA stability of TEA domain transcription factor 4 (TEAD4). In turn, TEAD4 served as a transcription factor that bound to the LINC01106 promoter and regulated its expression. Further assays indicated that LINC01106 promoted autophagy in LUAD cells by upregulating the expression of autophagy-related genes (ATGs). The silencing of LINC01106 in LUAD cells inhibited autophagy, and cell proliferation, and promoted apoptosis, which all were effectively reversed by ATG5 overexpression.

**Conclusions:**

Overall, LINC01106, transcriptionally activated by TEAD4, interacts with TAF15 to promote the stability of TEAD4 and upregulates the expression of ATGs, promoting malignancy of LUAD cells.

## Introduction

Lung cancer is the second most common cancer globally and the leading cause of cancer-related deaths [1]. It encompasses two histological subtypes: small cell lung cancer (SCLC) and non-small cell lung cancer (NSCLC) [2]. Among NSCLC, lung adenocarcinoma (LUAD) is the most prevalent subtype, accounting for 60% of all lung cancer cases [3]. Current treatment options for LUAD include surgical resection, chemotherapy, radiotherapy, and immunotherapy [4,5]. However, the prognosis for LUAD patients remains unsatisfactory due to frequent metastasis at advanced stages, with 5-year survival rates of 55% for localized lung cancer and only 5% for distant spread [6]. Consequently, there is a pressing need to develop novel targeting strategies to enhance the prognosis of LUAD patients.

Long noncoding RNAs (lncRNAs) are RNA transcripts longer than 200 nucleotides that lack protein-coding potential [7]. Abnormal lncRNA expression is linked to cell proliferation and autophagy in various cancers, including LUAD [8–12]. LINC01106 is an oncogenic lncRNA and exhibits an oncogenic function in multiple cancer types. For instance, it promotes bladder cancer cell growth, migration, and invasiveness through the regulation of ELK3 and HOXD8 [13,14]. In colorectal cancer, LINC01106 is highly expressed and enhances proliferation, migration, and stem-like characteristics of colorectal cancer cells via STAT 3 and LINC01106/Gli2 positive feedback loop [15,16]. Silencing of lncRNA LINC01106 targets miR-34a-5p and represses malignant behaviors of gastric cancer cells [17]. Gao et al. have highlighted the role of LINC01106 in suppressing endometrial cancer cell growth, migration, and invasiveness through its regulation of MET [18]. Additionally, Zhang et al. demonstrated in 2022 that LINC01106 silencing led to the inhibition of growth, migration, and invasiveness of non-small cell lung cancer (NSCLS) cells via miRNA-765 [19]. However, a crucial aspect that remains unclear is the involvement of LINC01106 in regulating autophagy and its impact on the progression of lung cancer. Therefore, it is imperative to investigate the biological function of LINC01106 in LUAD.

Autophagy has a dynamic role in either suppressing or promoting cancer progression, depending on the context and stage [20,21]. Autophagy-related proteins (ATGs) play a crucial role in autophagosome formation and collaborate with membrane lipids [22]. In lung cancer, ATG5 has been found to regulate malignant cell phenotypes, with high expression in lung squamous cell carcinoma tissues, and its silencing leads to autophagy suppression in these cells [23]. Recent research has demonstrated that circ-FOXM1, a transcription factor in promoting cancer, interacts with miR-149-5p to upregulate ATG5, promoting NSCLC cell growth and autophagy [24]. Another recent study revealed the involvement of Plasmacytoma Variant Translocation 1 (PVT1), a long non-coding RNA, in regulating autophagy through the miR-140-3p/ATG5 axis in lung cancer development [25].

TAF15, also known as TATA-box-binding protein-associated Factor 15, is an RNA/DNA binding protein. It plays critical roles in RNA splicing, mRNA transport, transcription, translation, and maintenance of genome integrity [26–28]. TAF15 has been implicated in multiple cancers, including breast cancer, gastric cancer, colorectal cancer, lung cancer, and myoepithelial tumors [29–33]. It interacts with lncRNAs and circRNAs to stabilize downstream target genes such as SMAD3 and SOCS3 [28,34]. TEAD4 is TEA domain-containing transcription factor, which is frequently overexpressed in diverse cancers, including ovarian, liver, colorectal, and gastric cancers [35–38].

Our study seeks to provide valuable insights into the role of LINC01106 in the progression of lung adenocarcinoma (LUAD). Previous studies have strongly suggested that TEAD4 plays a significant role in promoting LUAD by enhancing glycolysis and activating the ERK signaling pathway [39–41]. Furthermore, TEAD4 has been shown to promote metastasis in LUAD through miR-6839-3p [42]. However, the potential interplay between TEAD4, LINC01106, and ATG5 in promoting the progression of LUAD has remained unexplored. Therefore, our study aims to unravel the oncogenic impact of LINC01106 on LUAD progression and its interactions with TAF15, TEAD4, and ATG5. By shedding light on these complex relationships, our research will not only enhance our comprehension of the intricate molecular mechanisms underlying lung cancer but also open up new possibilities for LINC01106 as a potential therapeutic target for LUAD.

## Materials and Methods

### Cell culture

Human lung adenocarcinoma (LUAD) cell lines (A549, HCC827) and bronchial epithelial cells (BEAS-2B) were obtained from the American Type Culture Collection (ATCC, USA). BEAS-2B cells were cultured in Bronchial Epithelial Cell Growth Medium (BEGM, Lonza Group, Basel, Switzerland), while A549 cells were cultured in F-12K medium (Thermo Fisher Scientific, Fair Lawn, NJ, USA) supplemented with 10% FBS. HCC827 cells were cultured in Roswell Park Memorial Institute (RPMI) 1640 medium (Thermo Fisher Scientific, Fair Lawn, NJ, USA) supplemented with 10% FBS. All cell lines were maintained in a humidified incubator at 37°C with 5% CO_2_.

### Plasmid transfection

To inhibit LINC01006 and TEAD4 expression, two short hairpin RNAs (shRNAs) against LINC01006 (sh-LINC01106-1 and sh-LINC01106-2) and TEAD4 (sh-TEAD4-1, sh-TEAD4-2) along with non-targeting control shRNA (sh-NC) were designed and synthesized by Genechem (Shanghai, China). For sh-LINC01106-1, its forward sequence was 5′-CGAAGACACTTCAGTCAGTTT-3′ and its reverse sequence was 5′-AAACAAACTGACTGAAGTGTCTTC-3′. In case of sh-LINC01106-2, the forward sequence was 5′-CGTAGACTCATCAGTCTGATG-3′ and reverse sequence was 5′-GATCATACTGACGGATGTGACTACG-3′. The shRNA sequence used for TEAD4 were 5′–GGAACAAACUGUGCCUGAATT–3′, and the reverse were 5′–UUCAGGCACAGUUUGUUCCTT–3′. For shRNA control, the forward set used was 5′–UUCUCCGAACGUGUCACGUTT–3′, the reverse sequence was 5′–ACGUGACACGUUCGGAGAATT–3′. To overexpress TAF15 and ATG, we cloned complete coding sequences of these genes into lentivirus or pcDNA3.1 vectors. Empty lentivirus or pcDNA3.1 vectors were used as negative controls (pcDNA3.1/oe-NC). Cell transfection was performed using Lipofectamine 3000, and cells were incubated for 48 h.

### RNA fluorescence in situ hybridization (RNA-FISH)

FISH assays were employed to examine the expression of LINC01106 in LUAD cells and BEAS-2B cells using a FISH kit. The procedure involved seeding cells on slides, followed by fixation with 4% paraformaldehyde (PFA) for 20 min. Digestion was performed using proteinase K (20 μg/mL), and the slides were then incubated with prehybridization buffer at 40°C for 4 h. Hybridization was carried out using LINC01106 probes (RiboBio, Guangzhou, China). Cell nuclei were counterstained with 4′, 6-diamidino-2-phenylindole (DAPI). Images were captured using an inverted fluorescence microscope (Ti2-U, Tokyo, Japan).

### Real-time RT-qPCR

Total RNAs were extracted using TRIzol Reagent (Merck Millipore, Carrigtwohill, Ireland), followed by cDNA synthesis using a cDNA synthesis kit (Thermo Fisher Scientific, Fair Lawn, NJ, USA). Real-time PCR was performed using the Bio-Rad CFX 96 Real-time PCR system and TB Green Premix Ex Taq II kit. The RNA levels were determined using the 2^−ΔΔCt^ method, with normalization to GAPDH. The primer sequences can be found in Table 1.

**Table 1:** Primer for real-time RT-qPCR, detection of gene expression

Gene	Forward primer	Reverse primer
LINC01106	5′-GAACATGTGCTTGGTGTCG-3′	5′-CCAGTTTATATCATCAGCTATGCG-3′
TEAD4	5′-TCTGCACAGATCATCTCCG-3′	5′-AGGCTTCACATCATGGGAC-3′
TAF15	5′-GAGCAGCAAAGTTATTCTACCT-3′	5′-GCCCATAGCCAGAATAGCT-3′
ATG5	5′-GCTATTGATCCTGAAGATGGG-3′	5′-GATGTTCACTCAGCCACTG-3′
GAPDH	5′-TCATTTCCTGGTATGACAACGA-3′	5′-GTCTTACTCCTTGGAGGCC-3′

### Western blot

Total protein was extracted from LUAD cells using lysis buffer (Thermo Fisher Scientific, Fair Lawn, NJ, USA), and protein concentration was determined by BCA assays. The proteins were loaded onto a 12% SDS-PAGE gel and subsequently transferred to polyvinylidene difluoride (PVDF) membranes for 2 h. The membranes were then blocked with 5% skim milk powder and incubated with primary antibodies against TEAD4 (#PA5-21977, 1:1000, Thermo Fisher Scientific, Fair Lawn, NJ, USA), TAF15 (#PA5-27845, 1:2000, Thermo Fisher Scientific, Fair Lawn, NJ, USA), LC3-I/II (#ABC929, 1:500, Merck Millipore, Carrigtwohill, Ireland), and ATG5 (#PA1-46178, 1:500, Thermo Fisher Scientific, Fair Lawn, NJ, USA) overnight at 4°C, with GAPDH (Abcam, ab8245, 1:1000) serving as the loading control. HRP-conjugated secondary antibody (Cell signaling, CST#7074, 1:100) was then applied, and the membranes were incubated at room temperature in the dark for 2 h. After that, the membranes were washed at least three times (10 min per wash) in PBST. Protein visualization was performed using an enhanced chemiluminescence detection system.

### Dual-luciferase reporter assay

Luciferase reporter assay for luciferase activity was conducted to investigate the interaction between TEAD4 and the LINC01106 promoter. A fragment of the LINC01106 promoter containing a potential binding site for TEAD4 was inserted into the pGL3-basic vectors (Genecreate, Wuhan, China). LUAD cells were seeded in a 24-well plate at a density of 6 × 10^4^ cells per well and co-transfected with the pGL3-LINC01106 promoter construct and empty pGL3 vectors along with sh-TEAD4-1/-2 or sh-NC. After 48 h of transfection, the relative luciferase reporter activity was measured using the Dual-Luciferase Reporter Assay kit (Promega Corp., Madison, Wisconsin, USA), with Renilla luciferase activity serving as the internal control.

### DNA pull-down assay

A DNA pull-down assay was performed following previously described methods [43]. Oligo-probes synthesized by RiboBio were coupled to M-280 Dynabeads. The oligo-bead complex was then incubated with a blocking buffer at room temperature for 30 min. Subsequently, the DNA-bead complex was obtained using a magnetic column (Promega Corp., Madison, Wisconsin, USA). The DNA-binding proteins were eluted by using an elution buffer, and the eluted proteins were subjected to western blot analysis using an anti-TEAD4 antibody.

### Chromatin immunoprecipitation (CHIP) assay

The interaction between TEAD4 and LINC01106 was investigated using a ChIP assay kit. LUAD cells were cross-linked with 1% formaldehyde for ten minutes once they reached 80% confluency, followed by ultrasonic treatment. The samples were then centrifuged at 4°C and 13,000 rpm, and the resulting supernatant was collected and incubated with anti-TEAD4 antibody and anti-IgG (used as a negative control). Protein A/G magnetic beads were added to capture the antibody-chromatin complex, which was incubated overnight at 4°C. Subsequently, the bound DNA fragments were extracted and subjected to PCR analysis.

### RNA immunoprecipitation (RIP) assay

RIP (RNA immunoprecipitation) assays were performed following a previously described protocol [44]. LUAD cells were lysed using lysis buffer (Thermo Fisher Scientific, Fair Lawn, NJ, USA), and the remaining cell lysate was incubated with magnetic beads conjugated to anti-FUS and anti-TAF15 antibodies at 4°C. Anti-IgG was used as a negative control. After digestion with Proteinase K buffer, the immunoprecipitated RNA was extracted and subjected to RT-qPCR analysis.

### mRNA stability measurement

Transfected LUAD cells were treated with 2 mg/mL Actinomycin D (ActD, Merck Millipore, Carrigtwohill, Ireland) for 0, 3, 6, 9, and 12 h. RNAs were extracted from cells in each group, and the mRNA expression of TEAD4 and ATG5 was evaluated using RT-qPCR analysis.

### Clonogenic assay

Transfected LUAD cells were seeded in six-well plates at a density of 1000 cells/mL. The cells were cultured for 2 weeks at 37°C, followed by washing with PBS and fixation with 4% PFA for 15 min. Subsequently, the cells were stained with 0.1% crystal violet (Sigma-Aldrich, St. Louis, MO, USA) for 20 min, and the number of colonies was manually counted using microscopy.

### Cell counting Kit-8 (CCK-8) assay

Transfected LUAD cells were seeded in 96-well plates at a density of 5000 cells per well. After incubation for 0, 24, 48, and 72 h, CCK-8 solution (Beyotime, Haimen, China) was added to the wells and further incubated for 2 h. The optical density (OD) of LUAD cells at 450 nm was measured using a spectrophotometer.

### TUNEL assay

The terminal deoxynucleotidyl transferase dUTP nick end labeling (TUNEL) assay was performed to evaluate the apoptotic rate of LUAD cells. Briefly, LUAD cells from each group (2 × 10^5^) were plated on coverslips in a 24-well plate. Cell fixation was achieved using 4% paraformaldehyde (PFA), followed by treatment with methanol and 30% hydrogen peroxide (H_2_O_2_) at a ratio of 50:1 at room temperature for 30 min. The TUNEL apoptosis detection kit (Boster, San Jose, CA, USA) was used according to the manufacturer’s protocol to detect TUNEL-positive cells in each group. After incubation with DAB and staining with hematoxylin, the slides were dehydrated in a gradient of ethanol, mounted with a mounting medium, and observed under a microscope.

### JC-1 staining

The cell culture medium in each group was supplemented with 10 mg/mL of JC-1 dye (Life Technologies, Carlsbad, CA, USA) and incubated for 10 min at 37°C. Next, cells were washed and observed using a fluorescence microscope to visualize the red and green fluorescence channels.

### Immunofluorescence (IF)

LUAD cells were fixed using 4% PFA and permeabilized with 0.2% Triton X-100 (Beyotime, Haimen, China). Subsequently, cells were blocked with 10% normal goat serum and incubated overnight at 4°C with anti-LC3 antibody (#L8918, 2μg/mL, Merck Millipore, Carrigtwohill, Ireland). Following this, the cells were incubated with Goat Anti-Rabbit IgG, Cy3 conjugate, and DAPI was used for nucleus staining. The resulting images were captured using fluorescence microscopy (Olympus).

### mRFP-GFP-LC3 adenovirus infection

The mRFP-GFP-LC3 adenovirus (HanBio, Shanghai, China) was employed to monitor the autophagic flux in transfected LUAD cells according to the manufacturer’s instructions. LUAD cells from each group were infected with the mRFP-GFP-LC3 adenovirus for 48 h, and the autophagic flux was evaluated by quantifying the accumulation of red LC3 puncta using a fluorescence microscope.

### Development of xenograft

BALB/c nude mice (4–5 weeks old) were obtained from Vital River (Beijing, China) and were randomly divided into two groups: sh-NC and sh-LINC01106 (n = 5 mice per group). Transfected A549 cells (sh-NC and sh-LINC01106) at a concentration of 5 × 10^5^ cells/mL were subcutaneously injected into the right armpit of each mouse (0.2 mL/mouse). Tumor growth was monitored every 3 days until day 21. At the end of the experiment, the mice were sacrificed, and the tumors were collected and weighed. The animal study was conducted with the approval of the Ethics Committee of The First Affiliated Hospital of Anhui Medical University (EAP#667590881) and were in line with ARRIVE guidelines.

### Immunohistochemistry (IHC)

The mouse tumor tissues were fixed with 4% paraformaldehyde (PFA), dehydrated in a series of ethanol concentrations, embedded in paraffin, and sectioned into 5 μm slices. To block endogenous peroxidase activity, the sections were treated with a 3% hydrogen peroxide solution. After blocking with normal goat serum, the sections were incubated with a primary antibody against Ki67 (#ab16667, 1/200, Abcam) at 37°C for 2 h. Subsequently, the sections were incubated with an appropriate secondary antibody for 60 min at room temperature following three washes with PBS. The samples were then subjected to staining using 3,3′-diaminobenzidine (DAB) and hematoxylin, and images were captured using a microscope.

### Statistical analysis

Data analysis was performed using GraphPad Prism 8.0 software (GraphPad Software, La Jolla, CA, USA). All experimental data were presented as the mean ± standard deviation (SD). Two-group comparisons were assessed using Student’s *t-*test, while differences among multiple groups were evaluated using one-way ANOVA. A *p* value less than 0.05 was considered statistically significant.

## Results

### LINC01106 facilitates lung cancer tumor growth in vivo

**Figure 1:**
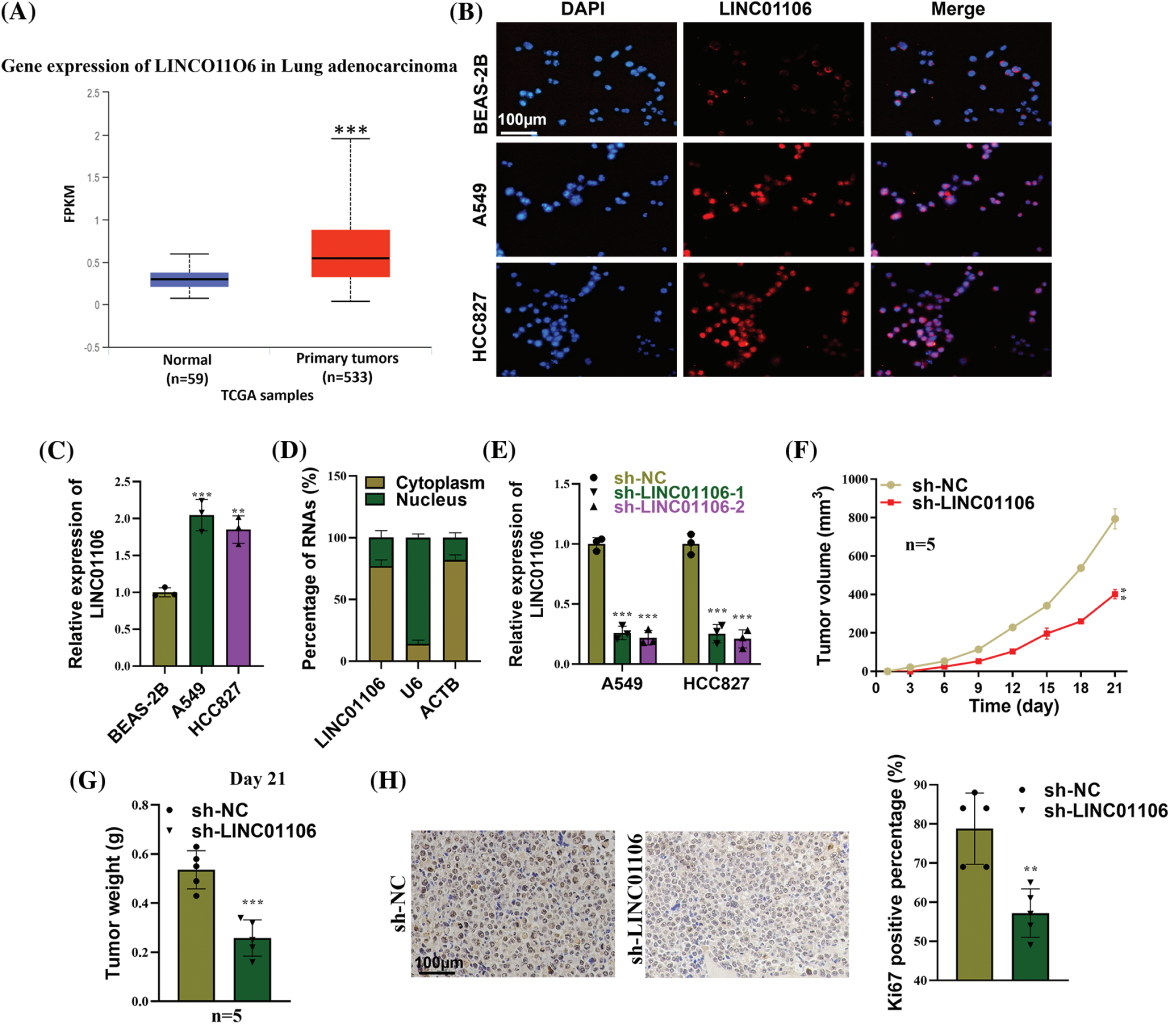
LINC01106 facilitates lung cancer tumor growth *in vivo*. (A) The expression profile of LINC01106 in LUAD tissues (n = 533) and normal tissues (n = 59) was analyzed using the UALCAN database (http://ualcan.path.uab.edu/). (B and C) The expression of Linc01106in LUAD cell lines (A549, HCC827) and non-cancerous BEAS-2B cells was detected using FISH assay and RT-qPCR (n = 3). (D) Subcellular fractionation assays were conducted to determine the subcellular location of LINC01106 (n = 3). (E) The efficiency of LINC01106 knockdown in LUAD cells was assessed using RT-qPCR analysis (n = 3). (F) The measurement of mouse tumor volume at specified time points in each group (n = 5). (G) Comparison of mouse tumor weight on day 21 between the sh-NC and sh-LINC01106 groups (n = 5). (H) Immunohistochemical (IHC) assays were performed to evaluate Ki67 protein expression in mouse tumor tissues from the indicated groups (n = 3). **p < 0.01, ***p < 0.001.

RNA-seq analysis conducted in a previous study revealed the upregulation of LINC01106 in lung cancer tissue samples [45]. Using UALCAN analysis, we further investigated the expression of LINC01106 in LUAD tissues and observed a high expression level (Fig. 1A). To validate these findings, FISH assays were performed, demonstrating the upregulation of LINC01106 in LUAD cell lines (A549, HCC827) compared to non-cancerous BEAS-2B cells (Fig. 1B). This result was consistent with the findings from RT-qPCR analysis (Fig. 1C). Additionally, subcellular fractionation assays confirmed the predominant localization of LINC01106 in the cell cytoplasm (Fig. 1D).

To elucidate the functional role of LINC01106, we employed a knockdown approach in LUAD cell lines. Transfection of sh-LINC01106-1/-2 led to a significant decrease in LUAD cell viability compared to the control group (Fig. 1E). *In vivo* experiments using xenograft tumor models demonstrated that injection of sh-LINC01106 resulted in the successful development of tumors with reduced volume and weight compared to those injected with sh-NC (Figs. 1F and 1G). Furthermore, immunohistochemical analysis revealed a significant decrease in Ki67 protein expression in mouse tumor tissues derived from sh-LINC01106 injected xenografts, suggesting that LINC01106 deficiency inhibits tumor cell proliferation *in vivo* (Fig. 1H).

### TEAD4 transcriptionally activates LINC01106

**Figure 2:**
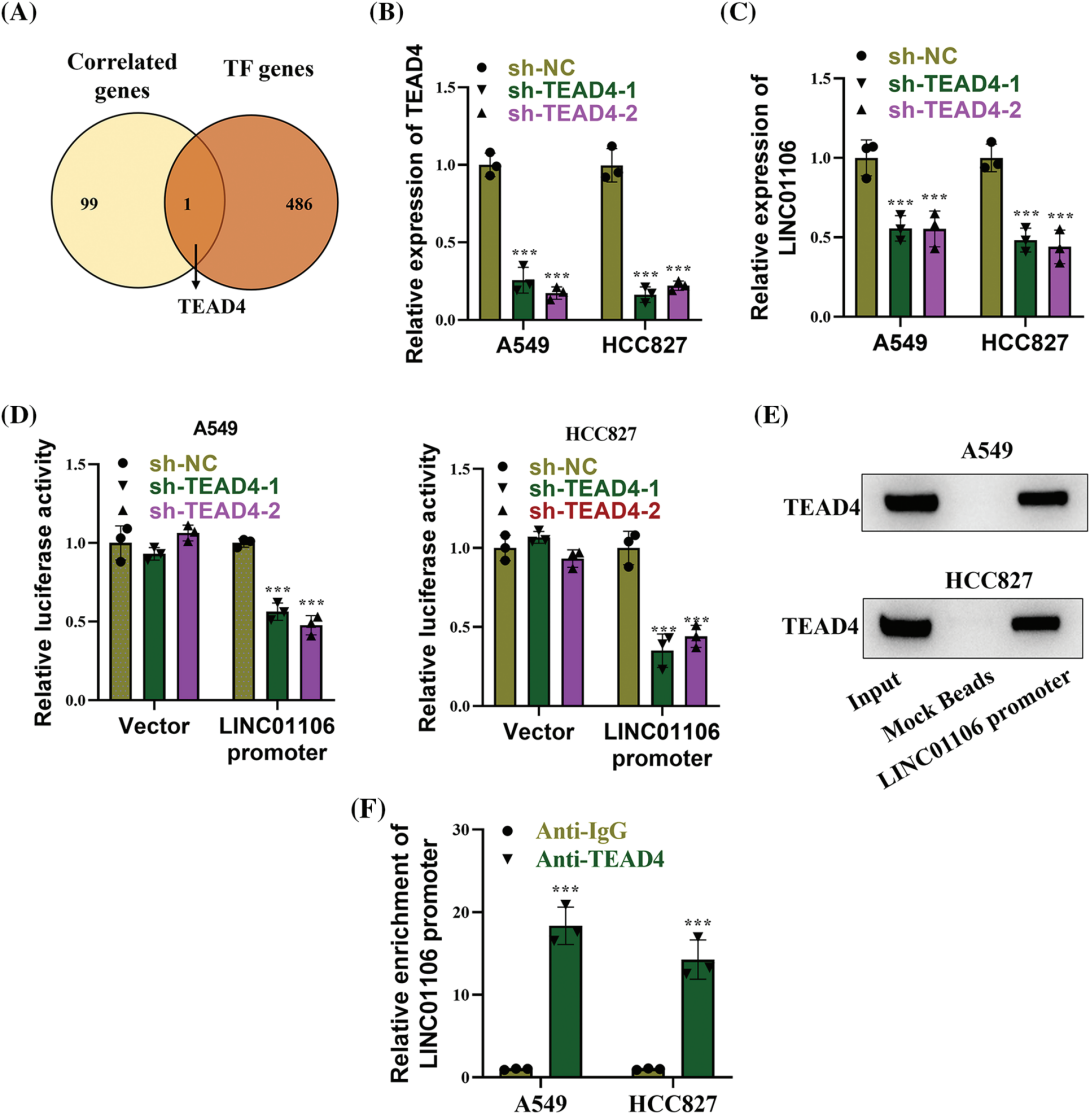
TEAD4 transcriptionally activates LINC01106. (A) Venn diagram illustrating the identification of potential transcription factors positively correlated with LINC01106 expression. This was achieved by intersecting the top 100 genes most significantly correlated with LINC01106 expression from the GEPIA database and the potential transcription factors from the Human TFDB database. (B) RT-qPCR analysis confirming the efficacy of TEAD4 knockdown in LUAD cells (n = 3). (C) RT-qPCR analysis assessing the expression of LINC01106 in LUAD cells transfected with sh-TEAD4-1/-2 (n = 3). (D) Dual-luciferase reporter assays conducted to investigate the interaction between TEAD4 and the LINC01106 promoter (n = 3). (E) DNA pull-down assay and Western blot analysis performed to examine the enrichment of TEAD4 in the complex pulled down by the LINC01106 promoter (n = 3). (F) ChIP assays employed to explore the binding between the LINC01106 promoter and TEAD4 in LUAD cells (n = 3). ***p < 0.001.

The potential transcription factors that exhibited a positive correlation with LINC01106 expression were identified by intersecting the top 100 genes significantly correlated with LINC01106 expression on the GEPIA database (http://gepia.cancer-pku.cn/) and the potential transcription factors on the Human TFDB database (http://bioinfo.life.hust.edu.cn/HumanTFDB#!/). Among them, only TEAD4 was selected, as depicted in the Venn diagram presented in Fig. 2A. We performed TEAD4 knockout experiments, and the efficacy of TEAD4 knockout in LUAD cells was confirmed through RT-qPCR analysis (Fig. 2B). Furthermore, we observed that silencing TEAD4 resulted in a reduction in LINC01106 expression in LUAD cells (Fig. 2C). Additionally, the luciferase reporter activity of the LINC01106 promoter was significantly decreased upon TEAD4 knockdown, indicating an interaction between the LINC01106 promoter and TEAD4 (Fig. 2D). Moreover, DNA pull-down assay and western blot analysis demonstrated abundant enrichment of TEAD4 in the complex formed with the LINC01106 promoter (Fig. 2E). To further validate this interaction, ChIP assays were conducted, which revealed a significant enrichment of the LINC01106 promoter in the precipitates of anti-TEAD4 (Fig. 2F). These findings strongly indicate that TEAD4 binds to the LINC01106 promoter and functions as a transcription factor for LINC01106.

### TTAF15 enhances the stability of TEAD4

**Figure 3:**
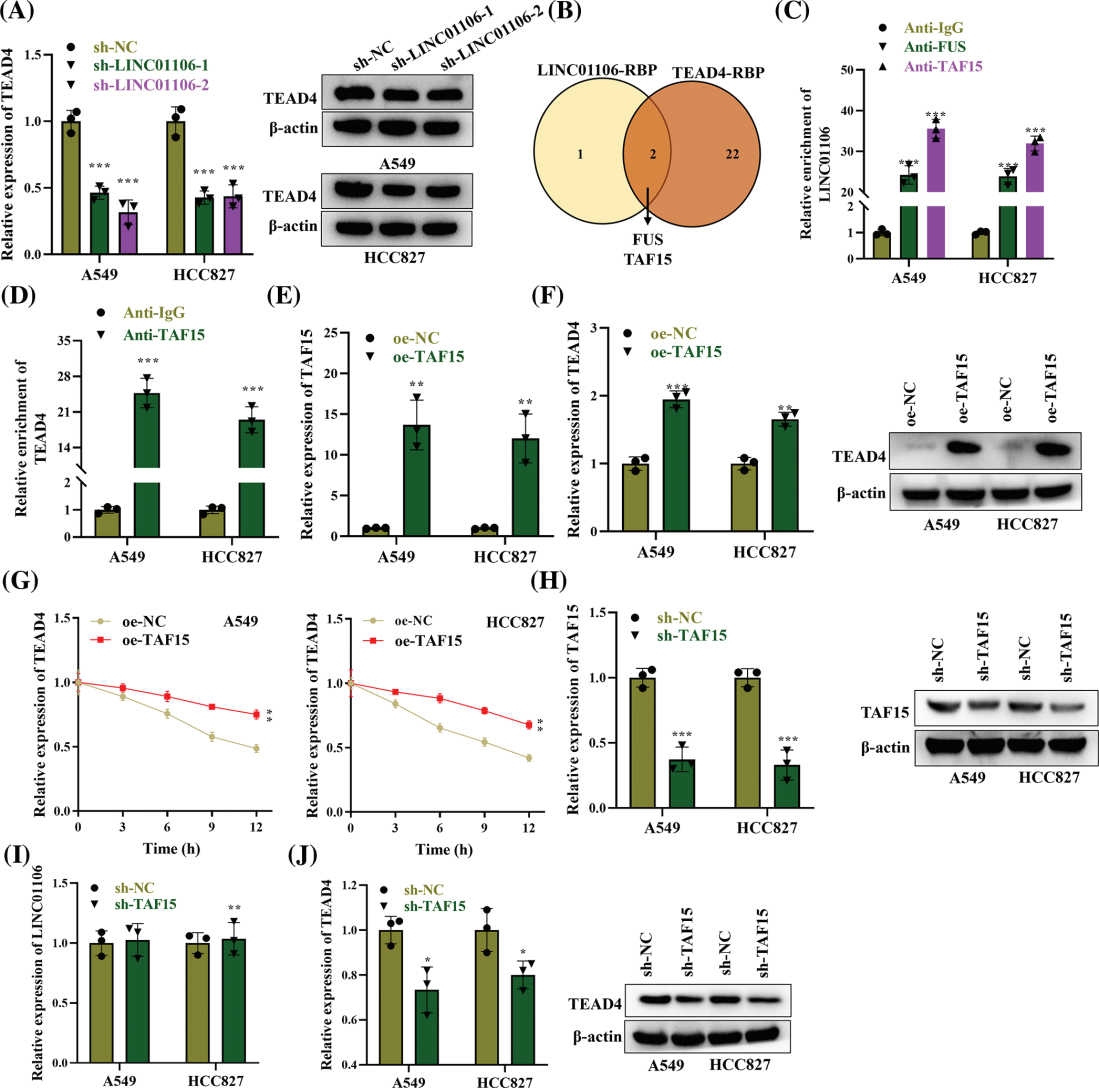
TAF15 enhances the stability of TEAD4. (A) The mRNA and protein expression of TEAD4 in LUAD cells transfected with sh-LINC01106-1/-2 was examined using RT-qPCR and western blot, respectively. β-actin was used as a loading control (n = 3). (B) Venn diagram showing the shared RNA-binding proteins (RBPs) for LINC01106 and TEAD4 based on the ENCORI database, under the screening condition of ClusterNumber ≥5. (C) RNA immunoprecipitation (RIP) assays were performed to evaluate the binding between LINC01106 and FUS or TAF15 in LUAD cells (n = 3). (D) RIP assays were conducted to detect the binding relation between TEAD4 and TAF15 in LUAD cells (n = 3). (E) RT-qPCR analysis was employed to examine the efficiency of TAF15 overexpression in LUAD cells (n = 3). (F) RT-qPCR analysis and western blot were used to detect TEAD4 mRNA and protein expression in LUAD cells after TAF15 overexpression. β-actin was used as a loading control (n = 3). (G) RT-qPCR analysis assessed TEAD4 mRNA levels in LUAD cells at different time points (0, 3, 6, 9, 12 h) after ActD treatment and indicated transfection (n = 3). (H) Knockdown efficacy of sh-TAF15 was validated by western blotting (n = 3). (I) RT-qPCR was conducted to assess influence of sh-TAF15 on LINC01106 expression (n = 3). (J) Effects of sh-TAF15 on TEAD4 expression were evaluated by western blotting (n = 3). *p < 0.05, **p < 0.01, ***p < 0.001.

We proceeded to investigate the effect of LINC01106 knockdown on TEAD4 expression. Our findings revealed that silencing LINC01106 resulted in a reduction in TEAD4 mRNA expression, and a similar pattern was observed in the protein level of TEAD4 (Fig. 3A). Given that lncRNAs can regulate gene expression by interacting with RNA-binding proteins (RBPs), we further examined the potential RBPs that could bind to both LINC01106 and TEAD4. Using the ENCORI database, we identified two RBPs, FUS and TAF15, that potentially interact with LINC01106 and TEAD4, based on a ClusterNumber ≥5 criterion (Fig. 3B). We specifically analyzed the binding of LINC01106 with FUS and TAF15 and found that LINC01106 exhibited the highest enrichment in the precipitates of anti-TAF15 in LUAD cells (Fig. 3C). Furthermore, we demonstrated the binding relationship between TEAD4 and TAF15 in LUAD cells (Fig. 3D). To investigate the functional role of TAF15, we overexpressed TAF15 in LUAD cells and confirmed its overexpression using RT-qPCR (Fig. 3E). Interestingly, the overexpression of TAF15 significantly increased TEAD4 mRNA and protein expression in LUAD cells, indicating a positive regulation of TEAD4 by TAF15 (Fig. 3F). TAF15 has been shown to enhance mRNA stability by binding to mRNAs [28]. Therefore, we examined the impact of TAF15 on TEAD4 mRNA stability. RT-qPCR analysis revealed that TEAD4 mRNA levels were significantly higher in the TAF15 overexpression group compared to the control group after treatment with actinomycin (ActD), a potent transcription inhibitor. This finding suggests that TAF15 enhances the stability of TEAD4 mRNA in LUAD cells (Fig. 3G). Furthermore, TAF15 was silenced using sh-TAF15, as evidenced by a significant reduction on TAF15 mRNA and protein levels (Fig. 3H) and silencing of TAF15 had no significant effect on LINC01106 expression (Fig. 3I). However, mRNA and protein levels of TEAD4 were inhibited by silencing TAF15 (Fig. 3J) in LUAD cells.

### LINC01106 recruits TAF15 to stabilize TEAD4

**Figure 4:**
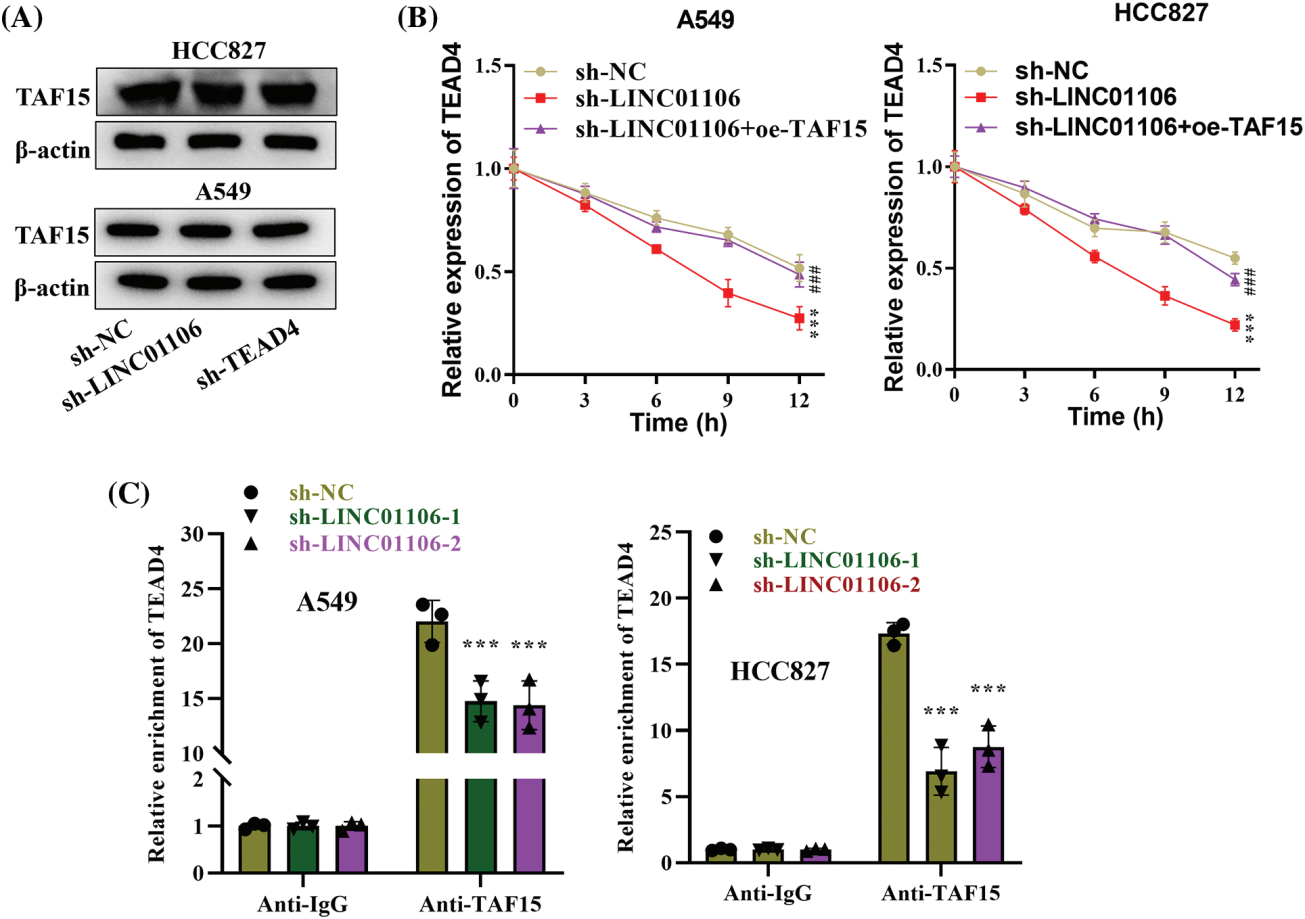
LINC01106 recruits TAF15 to stabilize TEAD4. (A) Protein expression of TAF15 was assessed by Western blotting in LUAD cells following LINC01106 or TEAD4 knockdown. β-actin was used as a loading control (n = 3). (B) mRNA expression of TEAD4 was measured using RT-qPCR analysis in LUAD cells after the indicated transfection (n = 3). (C) RIP assays were performed to examine the impact of LINC01106 silencing on the binding between TEAD4 and TAF15 in LUAD cells (n = 3). ***p < 0.001; ^###^*p* < 0.001.

We next investigated the impact of LINC01106 or TEAD4 knockdown on TAF15. Interestingly, we found that TAF15 protein levels remained unaffected in LINC01106 or TEAD4 silenced LUAD cells (Fig. 4A). However, at the mRNA level, RT-qPCR analysis demonstrated a significant decrease in the mRNA stability of TEAD4 upon silencing LINC01106, which was rescued by TAF15 overexpression, suggesting that LINC01106 regulates the mRNA stability of TEAD4 through the recruitment of TAF15 (Fig. 4B). Furthermore, RIP assays revealed a reduction in the enrichment of TEAD4 in anti-TAF15 precipitates after silencing LINC01106, indicating suppressed binding between TEAD4 and TAF15 (Fig. 4C). In conclusion, our findings suggest that LINC01106 plays a role in regulating TEAD4 mRNA stability by recruiting TAF15, highlighting a potential mechanism of gene regulation in LUAD cells.

### LINC01106 accelerates LUAD cell growth

**Figure 5:**
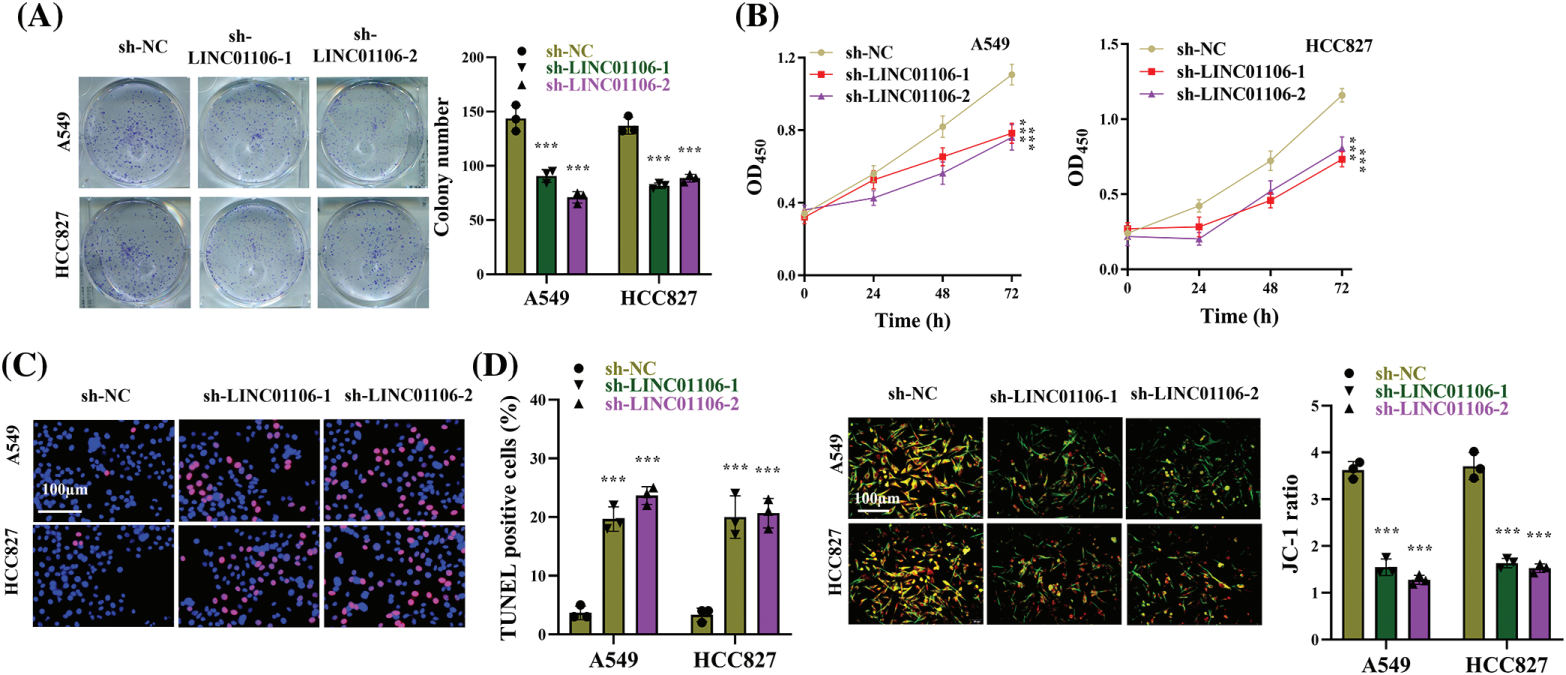
LINC01106 promotes LUAD cell growth *in vitro*. (A) Clonogenic assays were performed to assess the proliferation of LUAD cells following transfection with sh-LINC01106-1/-2 (n = 3). (B) Cell viability of LUAD cells in each group was measured using CCK-8 assays. (C) TUNEL assays were conducted to evaluate the extent of apoptosis in LUAD cells after the indicated transfection (n = 3). (D) JC-1 staining was performed to quantify the proportion of apoptotic cells (green) in LUAD cells (n = 3). ***p < 0.001.

Functional experiments were performed to investigate the impact of LINC01106 on LUAD cell malignancy. Our results demonstrated that LINC01106 knockdown significantly suppressed the colony-formation potential and viability of LUAD cells (Figs. 5A and 5B). Additionally, TUNEL assays revealed a significant increase in the proportion of TUNEL-positive LUAD cells following LINC01106 knockdown, indicating enhanced apoptosis (Fig. 5C). Furthermore, JC-1 staining confirmed that LINC01106 knockdown led to a notable increase in the proportion of apoptotic cells (green) (Fig. 5D). Collectively, these findings suggest that deficiency of LINC01106 inhibits LUAD cell growth and promotes apoptosis *in vitro*.

### LINC01106 regulates LUAD cell autophagy

**Figure 6:**
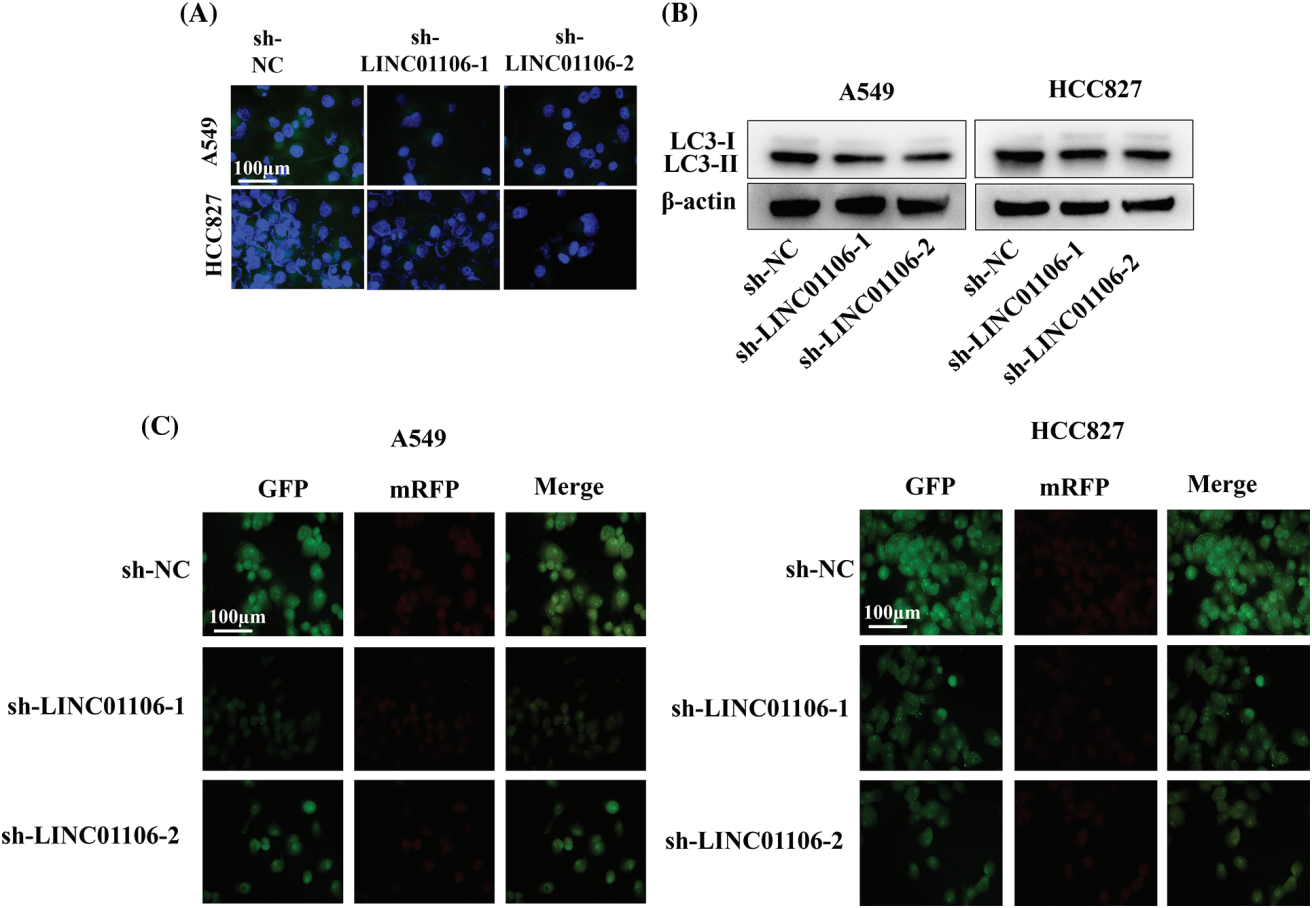
LINC01106 regulates autophagy in LUAD cells. (A) Immunofluorescence assays were performed to assess the expression of LC3 protein in LUAD cells following LINC01106 knockdown (n = 3). (B) Western blot analysis was conducted to measure the protein levels of LC3-I and LC3-II after silencing LINC01106 in LUAD cells. β-actin was used as a loading control (n = 3). (C) Autophagic flux in LUAD cells was visualized by transfecting cells with mRFP-GFP-LC3 adenovirus (n = 3).

We further investigated the impact of LINC01106 deficiency on autophagy in LUAD cells. Immunofluorescence (IF) staining showed a significant decrease in the expression of LC3 protein, a well-established autophagy marker, in LINC01106-silenced LUAD cells (Fig. 6A). Additionally, the ratio of LC3-II/LC3-I, which serves as an indicator of autophagic activity, was reduced upon LINC01106 silencing in LUAD cells (Fig. 6B). Furthermore, using mRFP-GFP-LC3 adenovirus transfection, we found that LINC01106 silencing led to a significant blockade of autophagic flux in LUAD cells (Fig. 6C). These results provide evidence that LINC01106 silencing exerts suppressive effects on autophagy in LUAD cells, suggesting its potential involvement in the regulation of autophagic processes in lung cancer.

### LINC01106 binds to TAF15 to upregulate ATG5

**Figure 7:**
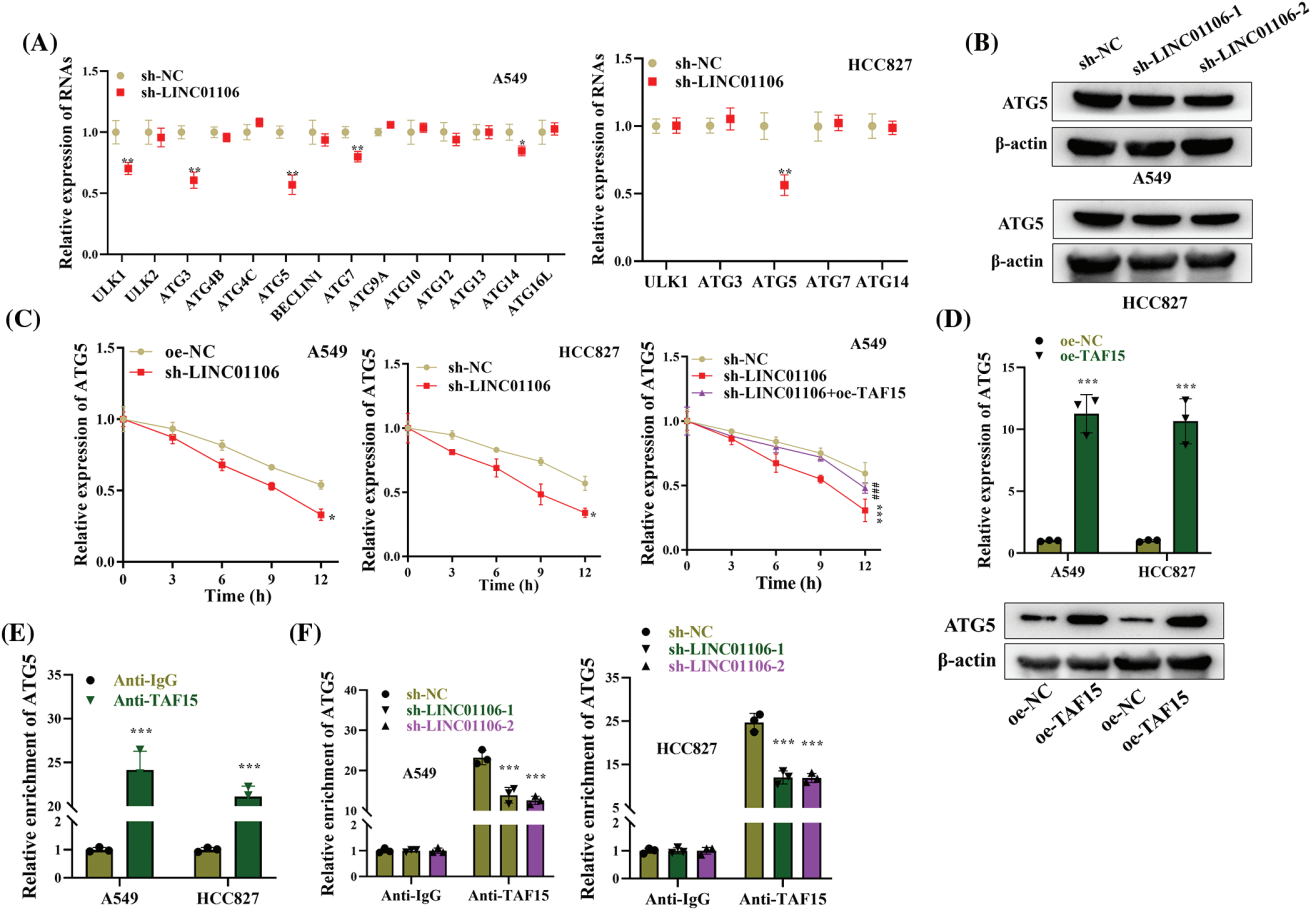
LINC01106 interacts with TAF15 to regulate ATG5 expression. (A) RT-qPCR analysis of ATG expression in LUAD cells after LINC01106 knockdown (n = 3). (B) Western blot analysis of ATG5 protein levels in LUAD cells after indicated transfections. β-actin is used as a loading control (n = 3). (C) RT-qPCR analysis of ATG5 mRNA expression and stability in LUAD cells treated with ActD and transfected with indicated constructs (n = 3). (D) RT-qPCR and Western blot analysis of ATG5 mRNA and protein levels after TAF15 overexpression in LUAD cells (n = 3). (E) RIP assays demonstrating the interaction between ATG5 and TAF15 in LUAD cells (n = 3). (F) RIP assays investigating the impact of LINC01106 knockdown on the binding between ATG5 and TAF15 in LUAD cells (n = 3). *p < 0.05, **p < 0.01, ***p < 0.001; ^###^*p* < 0.001.

ATGs are critically implicated in cell autophagy [46]. To understand the role of LINC01106 in the regulation of autophagy-related genes (ATGs), we investigated its impact on ATG protein levels in LUAD cells. Silencing LINC01106 led to a significant reduction in the levels of autophagic proteins including ULK1, ATG3, ATG5, ATG7, and ATG14s in A549 cells, while only ATG5 expression was decreased in HCC827 cells (Fig. 7A). Additionally, the protein expression of ATG5 was found to be decreased in LUAD cells after LINC01106 knockdown (Fig. 7B). Further analysis revealed that ActD treatment reduced the mRNA stability of ATG5 in LUAD cells with LINC01106 silencing, which was reversed by TAF15 overexpression knockdown (Fig. 7C). Interestingly, overexpression of TAF15 in LUAD cells resulted in elevated ATG5 mRNA expression and protein levels (Fig. 7D).

To confirm the interaction between TAF15 and ATG5, we conducted a RIP assay, which demonstrated the enrichment of ATG5 in the precipitates of anti-TAF15, indicating their binding in LUAD cells (Fig. 7E). Furthermore, we investigated the role of LINC01106 in the interaction between ATG5 and TAF15 by silencing LINC01106 in LUAD cells. This resulted in a significant decrease in ATG5 enrichment in the precipitates of anti-TAF15 compared to the sh-NC group in RIP assay, suggesting that LINC01106 silencing impaired the binding between ATG5 and TAF15 (Fig. 7F). These findings indicate that LINC01106 plays a role in regulating the interaction between ATG5 and TAF15 in LUAD cells.

### LINC01106 regulates LUAD cell growth by modulating ATG5

**Figure 8:**
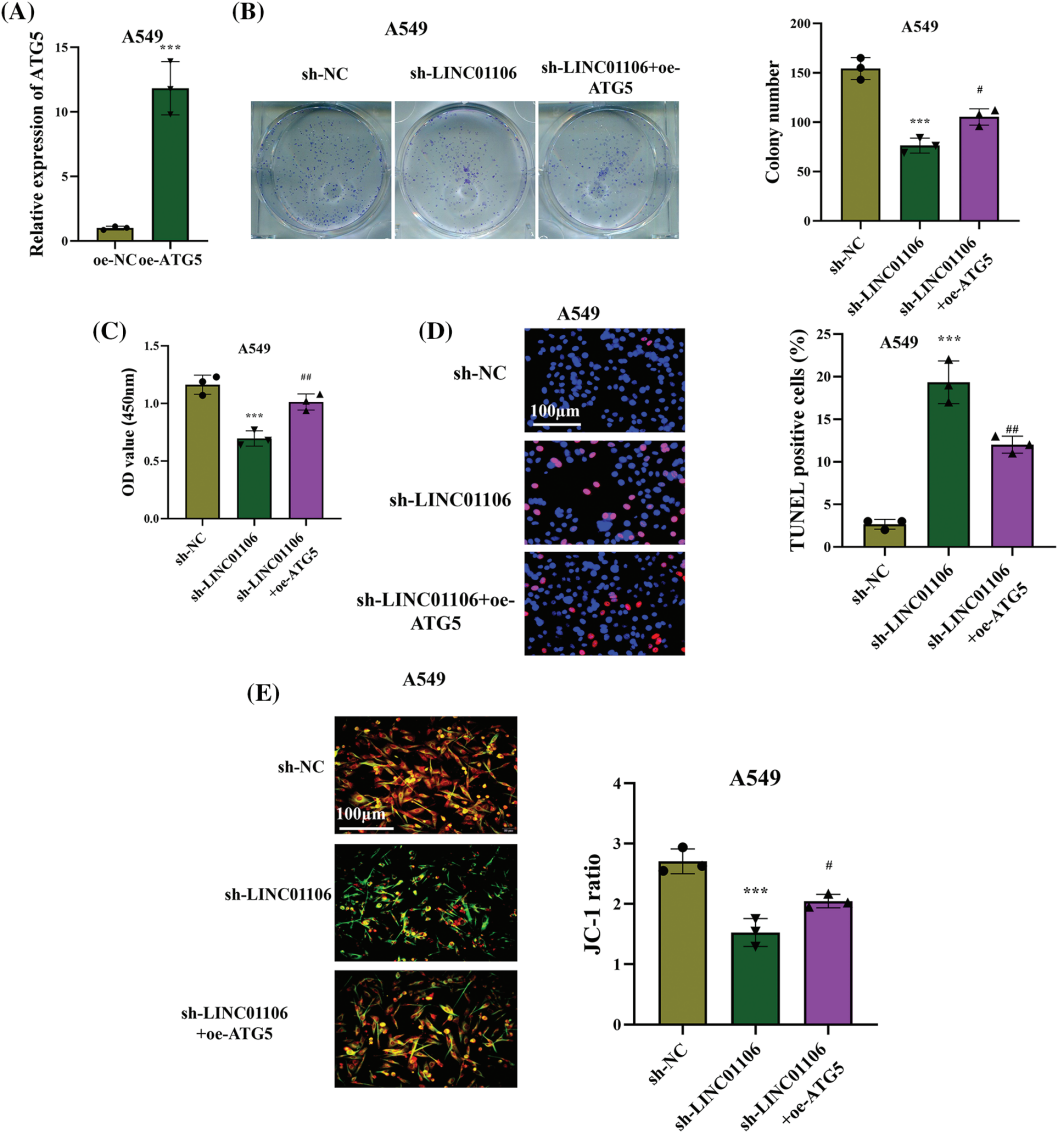
LINC01106 regulates LUAD cell growth by modulating ATG5. (A) RT-qPCR analysis confirms the overexpression efficacy of ATG5 in LUAD cells (n = 3). (B) Colony formation assays and (C) CCK-8 assays assess the proliferation of LUAD cells in each group (n = 3). (D) TUNEL assays and (E) JC-1 staining assays were performed to evaluate LUAD cell apoptosis after the indicated transfection (n = 3). Statistical significance is denoted by ***p < 0.001; ^#^*p* < 0.05, ^##^*p* < 0.01.

We finally elucidated the functional role of LINC01106 and its impact on LUAD cell behavior in presence of ATG5. The overexpression of ATG5 in LUAD cells was confirmed by RT-qPCR that showed a significant increase in expression levels of ATG5 in LUAD cells (Fig. 8A). Rescue assays showed that the reduction in colony number and cell proliferation induced by LINC01106 deficiency were effectively reversed by ATG5 overexpression (Figs. 8B and 8C). TUNEL assays demonstrated an increase in TUNEL-positive cells in the sh-LINC01106 group, which was rescued by ATG5 overexpression (Fig. 8D). Additionally, JC-1 staining revealed a significant reduction in the number of apoptotic LUAD cells after ATG5 overexpression, mitigating the effects of LINC01106 silencing (Fig. 8E). These findings collectively highlight the role of LINC01106 in enhancing LUAD cell malignancy through the upregulation of ATG5.

## Discussion

In our study, we observed that linc01106 plays a significant role in promoting LUAD development and its silencing had inhibitory effects on cancer development. Additionally, LINC01106 silencing exhibited suppressive effects on autophagy, while enhancing LUAD cell apoptosis.

TEAD4, a member of the TEA (Transcriptional enhanced associate) domain-containing transcription factors family including TEAD1, 2, 3, and 4, has been reported to possess oncogenic effects in various cancers [47]. Previous studies have highlighted the role of TEAD4 as an epigenetic regulator of gene transcription. For instance, TEAD4-activated MNX1-AS1 has been shown to facilitate gastric cancer progression through the EZH2/BTG2 and miR-6785-5p/BCL2 axes [48]. In lung cancer, TEAD4 is upregulated and promotes the proliferation, migration, and tumorigenesis of LUAD cells by transcriptionally activating and phosphorylating extracellular signal-regulated kinase (ERK) proteins [49]. Additionally, TEAD4 has been implicated in the migration, invasion, and epithelial-mesenchymal transition (EMT) process of LUAD cells [42]. In our study, we discovered that TEAD4 binds to the promoter region of LINC01106, functioning as a transcription factor to positively regulate LINC01106 expression in LUAD cells. Moreover, LINC01106 silencing exhibited suppressive effects on TEAD4 levels in LUAD cells.

Mounting evidence suggests that long non-coding RNAs (lncRNAs) play a crucial role in recruiting RNA-binding proteins (RBPs) to regulate downstream target gene expression [50]. For instance, a study unveiled the involvement of LINC00649 in recruiting TAF15 to stabilize MAPK6 expression, thereby promoting the progression of lung squamous cell carcinoma through activation of the mitogen-activated protein kinase (MAPK) signaling pathway [51]. TAF15 has also been implicated in binding to and facilitating the mRNA stabilization of SMAD3 [28]. In our study, we identified TAF15 as a common RBP for both linc01106 and TEAD4. We confirmed the interaction between TAF15 and linc01106 as well as TAF15 and TEAD4. Notably, TAF15 was found to positively regulate TEAD4 expression and enhance the mRNA stability of TEAD4 in LUAD cells. Moreover, we discovered that linc01106 recruited TAF15 to stabilize TEAD4 expression in LUAD cells, thereby exerting an additional layer of regulation.

Autophagy, a process that involves the degradation of intracellular components in lysosomes, plays a crucial role in maintaining cellular homeostasis and metabolism. Its role in cancer can be either cancer-promoting or tumor-suppressive, depending on the context [52–55]. Within autophagy, macroautophagy is regulated by a set of autophagy-related (ATG) proteins, and previous studies have highlighted the significance of ATG5-mediated autophagy in lung cancer progression [23–25,56]. In our study, we discovered that LINC01106 positively modulated ATG5 expression and contributed to its stabilization in LUAD cells. Additionally, we identified TAF15 as a binding partner for ATG5, which positively regulated both the mRNA and protein expression of ATG5. Moreover, LINC01106 was found to recruit TAF15 to modulate the mRNA stability of ATG5, resulting in its upregulation. The functional relevance of this interaction was confirmed through rescue assays, which demonstrated that LINC01106 regulated LUAD cell growth and apoptosis by modulating ATG5.

There are certain limitations of this study to consider. First, our findings are primarily based on *in vitro* experiments and xenograft models, which may not fully represent the complex *in vivo* tumor microenvironment. Further validation in sophisticated *in vivo* models is essential. Second, the clinical relevance of our results remains to be determined, and clinical studies are needed to evaluate the potential diagnostic and therapeutic applications. Last, a more in-depth mechanistic understanding of the molecular pathways involved in LINC01106-mediated effects on autophagy, proliferation, and apoptosis is needed to enhance the comprehensiveness of our research.

To build on our findings, future research should focus on detailed mechanistic investigations into the molecular pathways affected by LINC01106, TEAD4, and TAF15 could provide novel therapeutic targets. Additionally, the development of LINC01106, TEAD4, and ATG5 as potential biomarkers for LUAD diagnosis and prognosis prediction is an area worthy of exploration.

## Conclusions

In conclusion, LNIC01106 is transcriptionally activated by TEAD4 and forms a positive feedback loop with TAF15 to stabilize TEAD4. Furthermore, linc01106 interacts with TAF15 to upregulate ATG5, thereby promoting LUAD cell growth and autophagy while inhibiting apoptosis. Notably, silencing linc01106 also suppressed tumorigenesis in xenograft models indicating its involvement in progression of malignancy.

## Data Availability

The data and materials used in the present study are available from the corresponding authors upon reasonable request.
